# Prescribing practices of clozapine in India: Results of a opinion survey of psychiatrists

**DOI:** 10.4103/0019-5545.55097

**Published:** 2009

**Authors:** Amresh Shrivastava, Nilesh Shah

**Affiliations:** Department of Psychiatry, Mental Health Foundation of India (PRERANA Charitable Trust), The University of Western Ontario, and Lawson Health Research Institute, London, Ontario, Canada amresh.edu@gmail.com; 1 LTMG Hospital, University of Mumbai, Mumbai, India

Sir,

Clozapine has emerged as a gold standard for treatment resistant schizophrenia. It is also effective in a variety of clinical conditions. It is additionally approved for suicide in schizophrenia. However practice of clozapine therapy is complex due to its side effect profile, need for regular blood monitoring, continued clinical monitoring and requirements of logistical support system for the same. Indian conditions are diverse not only socio-economically but also in pattern of prescribing. Practice of psychiatry in India is therefore challenging. Internationally clozapine is a centrally monitored and guided therapy involving a number of agencies. The present report attempts to examine prescribing and monitoring practices of clozapine in Indian context.

Clozapine is an atypical antipsychotic, which is credited to have the distinction of bringing in the second revolution in psychopharmacology. It has been main focus of management for treatment-resistant schizophrenia. It has also provided a probe for the biological mechanism of schizophrenia. Many patients became functional with clozapine therapy and have returned to college and work.[1]

After several deaths due to agranulocytosis, the drug was approved with a condition of stringent blood monitoring, and this practice is strictly adhered to in each and every western countries.

Clinical practice in India does not have the benefit of a centralized blood monitoring system. The present study is aimed to explore the prescribing practice of clozapine in this context. Serum level monitoring is also not available in routine clinical practice. These constrains may change the practice of clozapine therapy.[2,3]

The study was done using a specifically prepared semi-structured performa containing 32 items and the questionnaire circulated to about 500 psychiatrists in India. A sizable number of posting was done in urban areas besides metro cities and the survey was voluntary. The responses received were analyzed.

A total of 117 responses were received in this study, 9.4% of the psychiatrists have reported using clozapine even before it was launched in Indian Market. Antipsychotic prescribing practices are influenced by several factors. Chong *et al.* noted that Japan had a high frequency of prescribing high doses and polypharmacy; Singapore had a high utilization of depot injections, while China had a higher prescription of clozapine. First-generation drugs were mainly for controlling aggressive behavior, while second-generation drugs were targeted at the alleviation of positive, negative psychotic symptoms as well as disruptive behavior in schizophrenia. The pattern of antipsychotic medication use varies in different countries and is likely to be influenced by the prevailing health-care system, the availability and cost of the drugs.[4]

Most of the psychiatrists using clozapine have an experience of more than 10 years (51%, between 10 and 20 yrs; 28% >20 years).

In the present study, 50% doctors have been using clozapine because they had one more option, and only 28% doctors felt that the knowledge of efficacy has guided their clinical decision.[5]

Further, 43% doctors felt that clozapine provides very good and excellent outcome. The majority opinion was that clozapine provided an efficacy from 40 to 70% in terms of symptom reduction.

Switching to clozapine is recommended under strict monitoring with a definite washout period either on outpatient or on inpatient basis.[6] However, in this study, only 39% preferred stopping ongoing antipsychotic before starting clozapine. A majority of them used the gradual introduction method, 41% preferred to start clozapine gradually, while gradually withdrawing the existing antipsychotic.

Eighty-four percent preferred to maintain patients on *single drug* clozapine as per best practices norm. While 16% preferred *combination* with conventional antipsychotics.[7] Augmentation of resistant cases by typical or atypical and several other molecules has been well reported.[8]

Dose initiation of clozapine in this study varied from 12.5 mg with 12% doctors, 75-100 mg with 7%, 25 mg per day with 58% and 50 mg per day with 23% doctors.

Average dose among the stabilized schizophrenia patients was reported to be 50 mg by 12%, 75–100 mg by 25%, 150 mg by 23%, 150–300 mg by 24% and more than 300 mg by 14% doctors. About 50% physicians reported effective dose between 150 and 300 mg per day. It is noteworthy that 37% physicians find effective response in symptoms at a dose of 50, 75, and 100 mg per day. Low-dose maintenance is an interesting finding, which needs confirmation and further study.

The blood monitoring practice is also different in India. In this study, 80% of the doctors preferred blood monitoring every week in the first month of the therapy and every month in the next 6 months. However, only 38% continued this practice of regular blood monitoring. Only 5% psychiatrists maintained the practice of once in 2 weeks monitoring after 1 year of treatment. Majority, i.e., 80% psychiatrist were comfortable with the view that monitoring should be done once in a week for the first month, and once in a month thereafter until 6 months and further depending upon “need.” Several cultural reasons may be responsible for this clinical opinion.

The common side effects observed were sialorrhea and sedation. And 14.5% psychiatrist reported being aware about agranulocytosis causing death as a side effect of clozapine usage, while 15% psychiatrists reported encountering any hematological emergency. A 32% doctors have reported the blood count to drop below 1000 and 15% below 2000 confirming the side effect of leukopoenia in the patients. No one reported any incidence where total count or absolute neutrophil count had dropped below 500, which is diagnostic prerequisite for agranulocytosis. This is an observation, which requires further scientific study. Preliminary evidence exists for the safety and efficacy of the concurrent administration of CLZ and ECT in CLZ-resistant patients.[9]

In this study 17% doctors never wanted clozapine to be used in combination with ECT, and 26% doctors felt that such a combination is a matter of routine while 30 % felt such a combination should be reserved for special circumstances only. Supplementing clozapine with a course of bilateral ECT appears to be safe and is effective; however, its long-term impact in incompletely responsive patients is not fully elucidated.[10]

We found that 59% prescribes had observed incident of seizures as a side effect in their patients. To manage the same, 39% of the doctor's reduced clozapine dosage, 39% used an anticonvulsant drug, and 24% stopped clozapine therapy completely in seizure occurrence.

In the present study, major indication has been treatment-resistant schizophrenia, among 91% indications. The usage of clozapine in other condition emerges strongly e.g., 15.4% have reported using clozapine in the first episode of psychosis, and 38% have started using it in schizoaffective and 30% in schizomania, 32% for Parkinson's with psychosis, 24% for movement disorder,[11] and 12% in childhood onset schizophrenia.

The study reveals that blood monitoring practice is unique and satisfactory as seen from low rates of life-threatening side effects. As per the survey, no incident of either agranulocytosis or any death by such a complication was reported.

The study concludes that practice and usage of clozapine varies in Indian culture and the variation has no negative therapeutic effects.
